# A case study of patient and public involvement in sepsis genomics research

**DOI:** 10.1186/s40900-026-00962-w

**Published:** 2026-09-15

**Authors:** Angeline Lee, Hanyu Qin, Kiki Cano-Gamez, Julian C. Knight

**Affiliations:** 1 Centre for Human Genetics, Nuffield Department of Medicine, University of Oxford, Oxford, UK; 2 Chinese Academy of Medical Sciences Oxford Institute, University of Oxford, Oxford, UK; 3 Department of Clinical and Biomedical Sciences, University of Exeter, Exeter, UK

**Keywords:** Patient and public involvement, Sepsis, Discovery research, Patient engagement, Genomics

## Abstract

**Background:**

Patient and public involvement (PPI) in research has been shown to be integral to good research practices, ensuring research is relevant to the population it serves and appropriately conducted. However, there are few examples in the literature of patient and public involvement in discovery translational research using patient samples from non-interventional studies. The aim of this case study is to report our group’s experience incorporating PPI into such work involving sepsis genomics research over a year, challenges we faced, and how we overcame them.

**Methods:**

We first reviewed the literature for successful PPI practices, asked for advice from other experienced groups at our institution, engaged interested groups and individuals, decided on the key aims of the PPI group, and developed materials to summarise our research and the knowledge gaps within sepsis. We recruited fourteen partners with lived experience of sepsis through a variety of sources to join our PPI panel and built a relationship with them by having regular meetings, and nominating a link researcher in the group who would be their port of call for any questions or updates. The partners met with the researchers online using a commercial virtual communication platform, and were sent surveys at different points during the process to assess partner satisfaction with the process. We followed the plan-do-study-act method for quality improvement.

**Results:**

The PPI partners helped shape the direction of research investigation for the group. They joined research grant applications, supported preparation of plain language summaries and research participant information leaflets to be given to patients and families. They helped researchers improve the conduct of the clinical aspect of studies that involved collecting patient samples in the long term. The Patient and Public partners reported high levels of satisfaction joining the programme and received some educational information about the research process and sepsis research, as well as remuneration for their time.

**Conclusions:**

Patient and public involvement in discovery translational research is feasible and can be very useful and rewarding for both the researchers and patient and public partners involved. A coordinated strategy including engagement of interested groups and individuals, clear directions for the PPI group, and a named link researcher was essential for success.

## Plain language summary

When scientists do research, it’s important that they listen to patients and the public. This helps make sure the research is helpful and done the right way. But this doesn’t happen often in “discovery” research—research done in the lab before it’s tested on people. Our team wanted to include patients and the public in our lab research about sepsis (a serious illness caused by infection). We started by reading what others had done, asked for advice, and made simple materials to explain our work. Then, we invited 14 people to join our patient and public panel. We talked with them regularly online, gave them surveys to check how things were going, and had one researcher act as their main contact. These panel members helped guide our research. They gave ideas about what questions we should study and helped us write easy-to-understand information for patients and families. They also helped us improve how we collect samples from patients. The panel members said they enjoyed being part of the project. They learned more about science and sepsis and were paid for their time. In the end, we found that it is possible and helpful to involve the public in early science research. Having clear goals, good communication, and a main contact person helped make it all work well.

## Background

There is growing awareness of the critical importance of patient and public involvement in research (PPI). PPI is defined by the National Institute for Health Research (NIHR) as ‘research being carried out with or by the members of the public, rather than ‘to’, ‘about’ or ‘for’ them.’ Members of the public include patients, potential patients, carers and people who use health and social care services, as well as people from organisations that represent people who use services [1, 2].

PPI is fundamental to ensuring that research is relevant and in the best interests of the patient groups involved and the wider society. There is a growing evidence base showing that PPI in research across various fields reduces research waste [3], improves study quality [3, 4] and makes research more relevant [4]. Beyond improving the relevance and quality of research, patient and public involvement is increasingly viewed as an ethical imperative. Individuals and communities affected by research have a legitimate right to contribute to decisions about research priorities, design and conduct, reflecting principles of democratic accountability, citizenship and justice [5, 6].

Sepsis is life-threatening organ dysfunction caused by a dysregulated (maladaptive) host response to an infection [7]. The condition is complex, arising in different forms from various pathogens encompassing bacteria, viruses, parasites and fungi. Clinical symptoms can vary, and it can lead to organ failure, tissue damage and death [7]. Sepsis is common, causing 166 million cases and 21.4 million deaths globally in 2021, which represented 31.5% of all global deaths that year [8]. However, patient and public awareness about sepsis is limited, despite programmes to increase understanding about the condition [9]. This could be because of varying definitions of sepsis, and the heterogenous, short-term nature of the acute condition that can be linked to multiple infections.

**Fig. 1:**
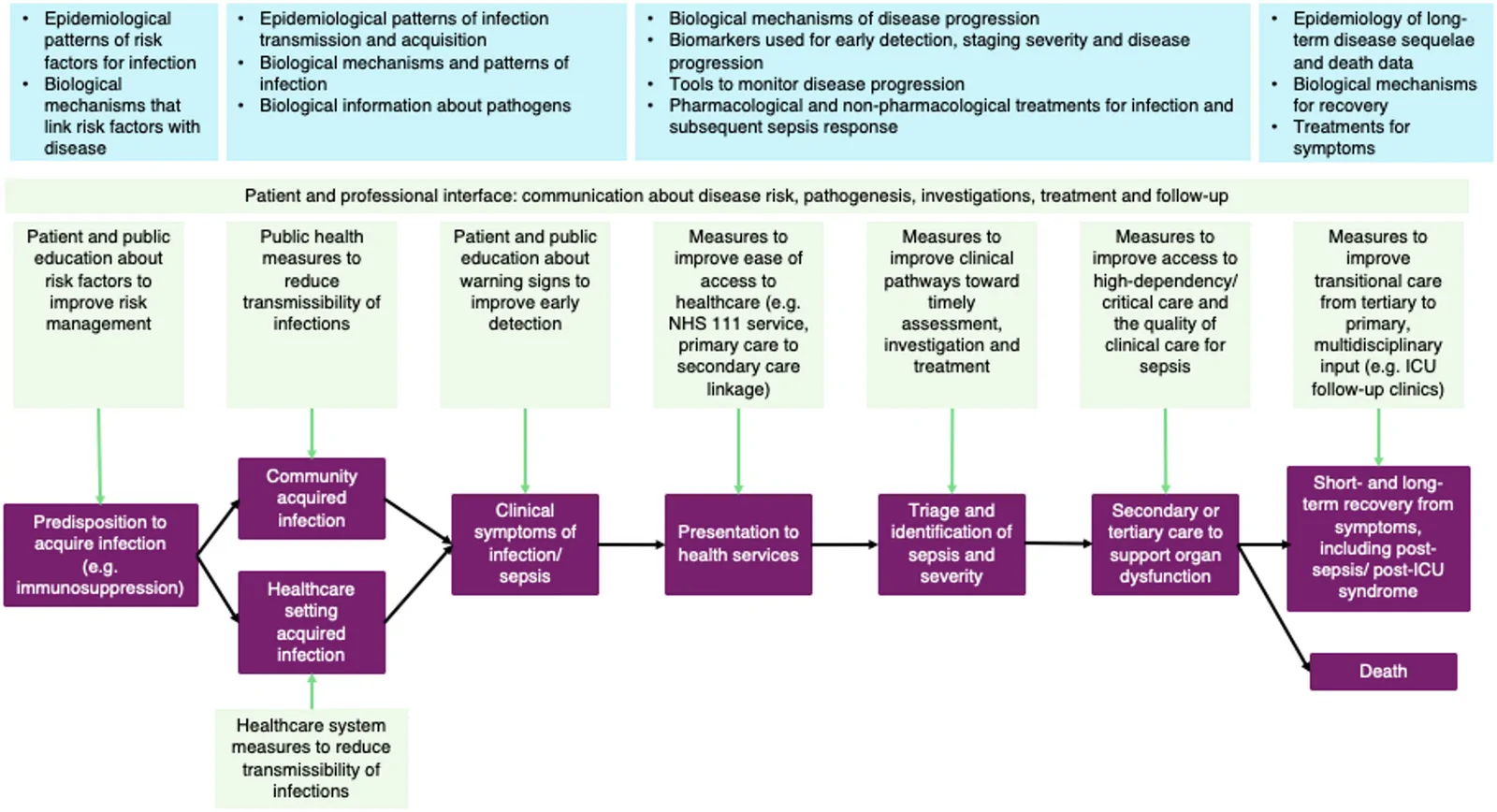
Schematic diagram of patient experience pathways in sepsis (purple boxes), patient-facing interventions and research (green boxes), and discovery research (blue boxes, applicable to the sepsis research of our group into biological mechanisms and precision medicine approaches using genomic, immunological and molecular approaches) involved in understanding and improving outcomes of this disease. Each green and blue box represents an opportunity for PPI in research

Existing evidence showed many PPI projects in a variety of research areas from clinical trials [10] to health economics [11], but there is relatively little evidence of PPI work in discovery translational research settings (also referred to as preclinical research) [12, 13]. Sepsis is a complex condition, and research about the disease and clinical course can encompass a range of areas (Fig. 1). There is scope for PPI involvement at every step (Fig. 1), and successful projects have been run to improve infection control guidelines in healthcare settings [14], and diagnosis and treatment of infection in critical care [15].

Researchers within our group use laboratory and bioinformatic techniques to understand how variation between individuals leads to differing immune responses to infection, and how this can contribute to susceptibility to other infectious, inflammatory and autoimmune diseases [16]. Many of our experiments utilise clinical samples and other relevant clinical information obtained from patients with sepsis from acute hospitals. This has allowed us to advance a precision medicine approach to sepsis, including subgroups of patients within sepsis with specific mechanisms of disease, and new opportunities for sepsis diagnostics and therapies.

In late 2021, we sought to expand the use of PPI to help guide our future research. The Surviving Sepsis campaign has identified a number of research priorities for sepsis research [17], many of which could be addressed by the experience and skillset within our research group. Existing evidence showed that recruitment of PPI members for infection-related studies was feasible but can be challenging to operationalise [18] with many sepsis patients remaining unwell after hospital discharge. Our research group unanimously felt that it would be important for patients to help guide the researchers toward the line of investigation that was most pertinent to those suffering from the condition.

In this article, we aim to report our experience conducting PPI in adult sepsis genomics research at the Centre for Human Genetics in the University of Oxford. We show how PPI can inform discovery translational research work using patient samples from non-interventional studies, and highlight benefits and challenges in performing high quality PPI in this setting.

## Methods

### Background preparation

**Fig. 2:**
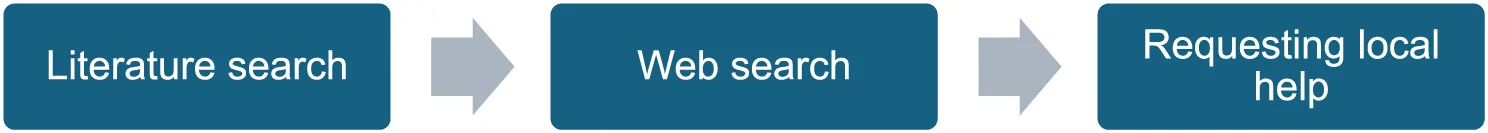
Background preparation for PPI initiative

Figure 2 shows a process map of background work conducted to start planning for our PPI initiative. A literature search was conducted across PubMed and Medline using terms “Patient and public involvement”, “PPI”, “PPIE”, “Public engagement”, and “Patient engagement” to understand good practices for PPI. Studies involving PPI in a critical care or infectious disease setting, and studies involving PPI in discovery translational research were analysed to determine a strategy for PPI recruitment and conducting meetings and potential outputs from PPI work. A web-based search was conducted to identify useful resources, which included the NIHR’s INVOLVE toolkit [2]. The University of Oxford’s Patient and Public Involvement and Engagement (PPIE) department, the local NIHR Research Design Service and other clinically based research groups who had previously used PPI were able to provide useful advice.

**Fig. 3:**
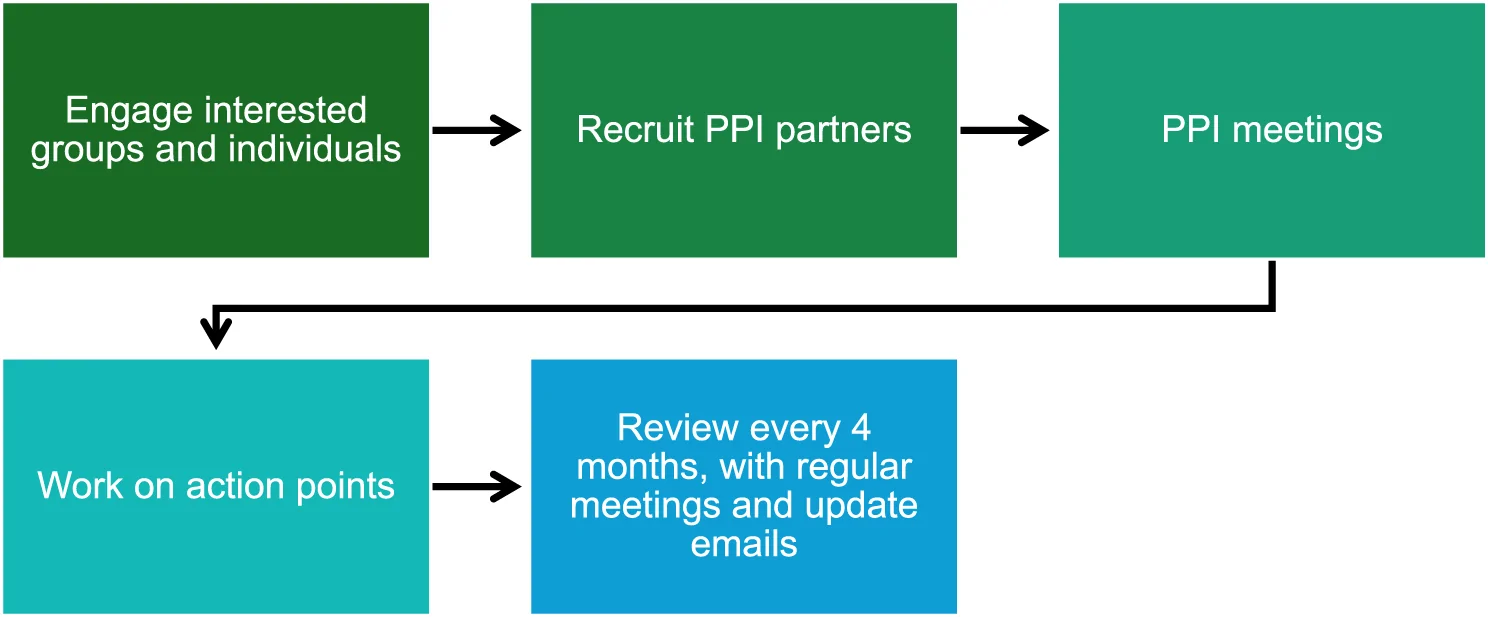
Process map for PPI initiative

Figure 3 shows a flow-diagram of the final PPI initiative designed based on the knowledge obtained during the background preparation process. We followed the plan-do-study-act method for quality improvement [19].

### Engaging interested groups and individuals

The primary PPI lead (AL) and Principal Investigator JK began the project by engaging relevant groups and individuals involved in the group’s activities (including patients, researchers, clinicians, patient organisations, charities). This included advertising the PPI project by presentations during laboratory meetings, email summaries and group discussions on Microsoft Teams (www.microsoft.com), and approaching relevant individuals. The engagement process consisted of a brief description of PPI and its relevance to the group, and detailed discussions to determine the specific goals of our PPI initiative, practicalities including methods of remuneration to partners (‘partners’ defined here as individuals with lived experience of sepsis or caring for those with sepsis who were part of the PPI initiative and joined the online meetings, comprising patients and carers; ‘participants’ refers to acutely ill patients recruited to the sepsis research study) and safety considerations (e.g. how to respond if a clinical question were to be raised with a researcher), and perspectives on the strengths and challenges of our proposal. Each partner was offered £25 per hour for their time for their involvement in the first year of the project as a thank you remuneration for a contributor’s time, skills and expertise, in line with NIHR guidance.

The result was a defined mode of operation with a clear definition of what the roles and responsibilities of the PPI partners would involve in the short, medium and long term, and how they would be involved in the group’s work.

### Recruiting patient and public partners

An email advertisement for the group’s PPI work was created and sent out to intensive care unit follow-up services in the Thames Valley area, the University of Oxford PPIE mailing list and departmental social media sites, and to major charities for sepsis, the UK Sepsis Trust (https://sepsistrust.org/) and Sepsis Research (FEAT) (https://sepsisresearch.org.uk/). The advertisement contained a brief description of what PPI involved, the group’s work and practical information such as remuneration rates. All interested individuals were then invited for an initial one-on-one meeting with AL and received further information about the group’s work and the patient and public partner role before confirming their involvement. They also completed an anonymous Google Forms survey (https://surveys.google.com) on their reasons for joining the PPI group before the first PPI meeting. We aimed for a minimum of 10 participating partners.

### PPI meetings

All patient and public partners and the research group were invited for a first PPI meeting on a date agreed via Doodle poll (www.doodle.com/en/online-survey/), with email reminders before the meeting and contact details for technical support if required. All meetings were conducted online, typically for one hour. An agenda for the meeting was disseminated to all attendees before the meeting, setting out the topics or questions for panel members to consider in advance. The research team were re-briefed on the project before the meeting and a member of the research team was designated to take minutes of the meeting. Organisation of the practicalities of the meeting were informed by the survey results.

Each meeting was conducted with careful facilitation to ensure that all partners were given a chance to get to know each other, have equal opportunities to speak and contribute as a focus group, and were not overwhelmed with information. Icebreaking questions, breakout rooms and short breaks were used effectively during the hour-long meeting.

After the meeting, minutes and action points arising were prepared by AL and disseminated to all attendees with clear description of action points and outcomes of the meeting, and information about next steps.

Six meetings were conducted in this manner over the course of one year (approximately 1 meeting every 2 months 2021/2022) in between working on action points. Email updates were sent out in between meetings to keep partners informed about progress. A PPI section was set up within the group’s research website. The final PPI partner and researcher satisfaction was assessed by emailing each participant individually and asking for feedback and whether they would be keen to continue participation for the next year, adopting a personalised approach and asking open ended questions to help develop the PPI programme further.

## Results

Fourteen patient and public partners were recruited within 2 weeks of the initial advertisement. Patient and public partners represented a diverse group of patients and carers of different ages, gender, ethnicity and geographical location.

The first year of the PPI initiative led to several important outputs for our research group. These included a PPI meeting where we sought input in setting research priorities for future research. Patient and public partners prioritised understanding why some individuals were at risk of sepsis, developing better treatments for the acute disease and improving the care and outcomes of sepsis survivors as partner research priorities. A further important outcome was recruitment of PPI members into a steering group for future research including grant applications, and reviewing plain English summaries of proposed research (shared virtually for comment). The research partners input led to improved recruitment processes for our clinical studies including the development of user-friendly patient information leaflets and understanding of potential barriers to recruitment and how this might be overcome. A further important outcome was prioritising appropriate allocation of a PPI budget for all future group research.

The clinical experience of sepsis by the PPI group provided helpful insights and included feelings of uncertainty, being too ill to take in any information, themselves and their families trying to process a large amount of information about their condition and the disease, and being in a rapidly evolving situation. We had many discussions about the practicalities of recruiting participants to studies like ours in this setting, and the PPI group were able to help us develop patient information leaflets for our study that conveyed information about the study more clearly, alongside signposts toward useful resources to support patients through their illness, such as links to the Sepsis Trust. Long-term follow-up sample acquisition was also an interesting area of discussion, and the PPI group were able to provide advice about the reasons why participants often became lost to follow-up after hospital discharge, including difficulties mobilising, sleep disruption, and inability to drive while recovering from their illness.

**Fig. 4:**
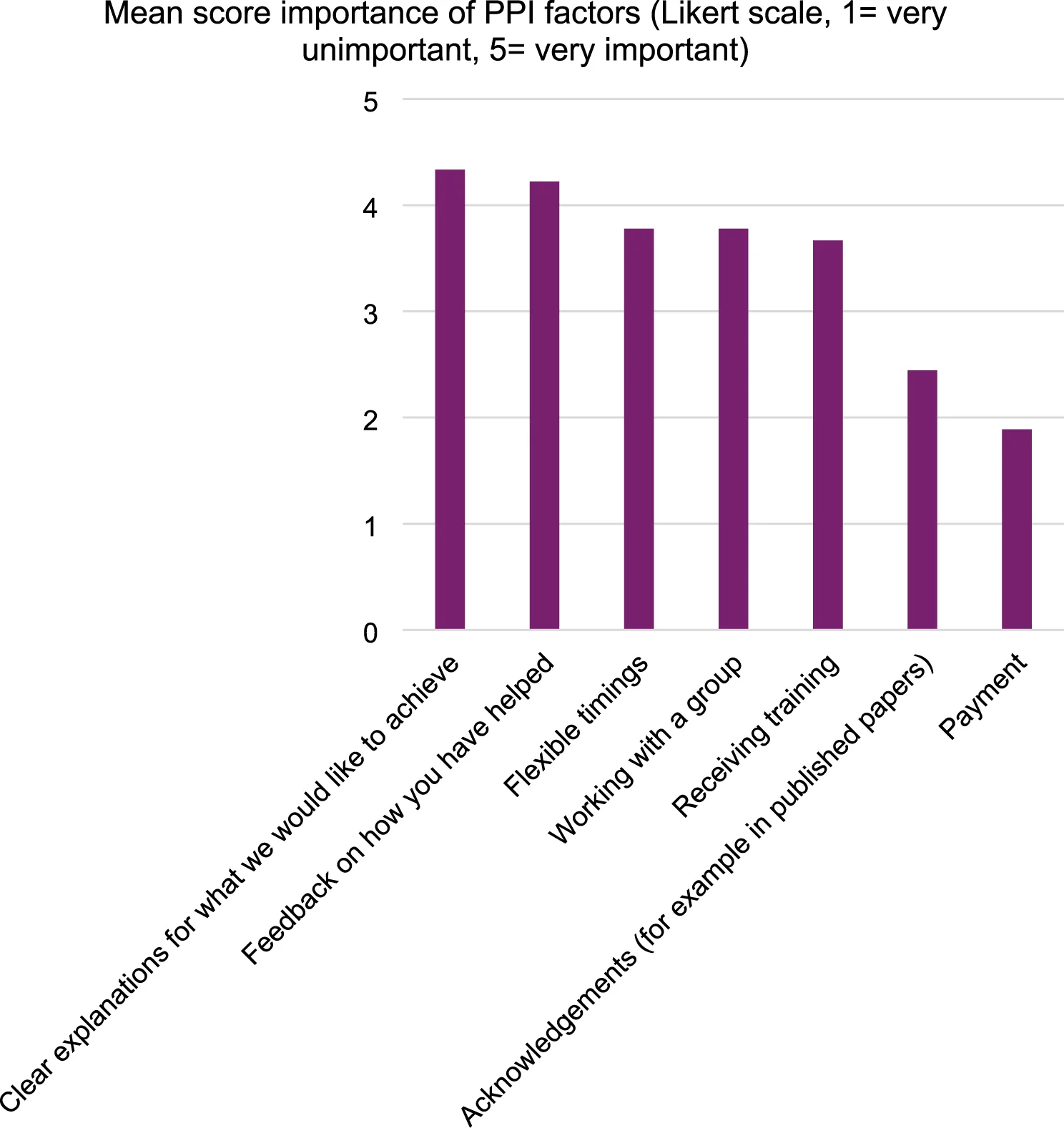
Mean score rating importance of PPI factors. Nine out of fourteen partners responded to the survey

We sought to understand partners motivation for being involved and what was important to them in this process. Nine out of 14 partners responded to a Google survey about the PPI involvement after being recruited into the group and before the first PPI meeting. Partners ranked the importance of different aspects of PPI (Fig. 4) and highlighted the importance of using plain language and giving partners feedback on the results of their involvement. Remuneration was universally ranked as unimportant or very unimportant. Most of the partners declined to be reimbursed and asked that the money be donated to sepsis research charities instead. White-space comments about the importance of flexible timings and clear explanations for what was needed guided the organisation and planning for PPI meetings and activities.

Several patient and public partners highlighted the importance of considering their condition as sepsis survivors when providing information, as many continued to be very symptomatic and unwell long after their initial illness and would struggle to take on a lot of information or concentrate for prolonged periods. These comments guided information dissemination – emails were purposely kept short and written information was provided in small, manageable chunks over time for reference.

**Fig. 5:**
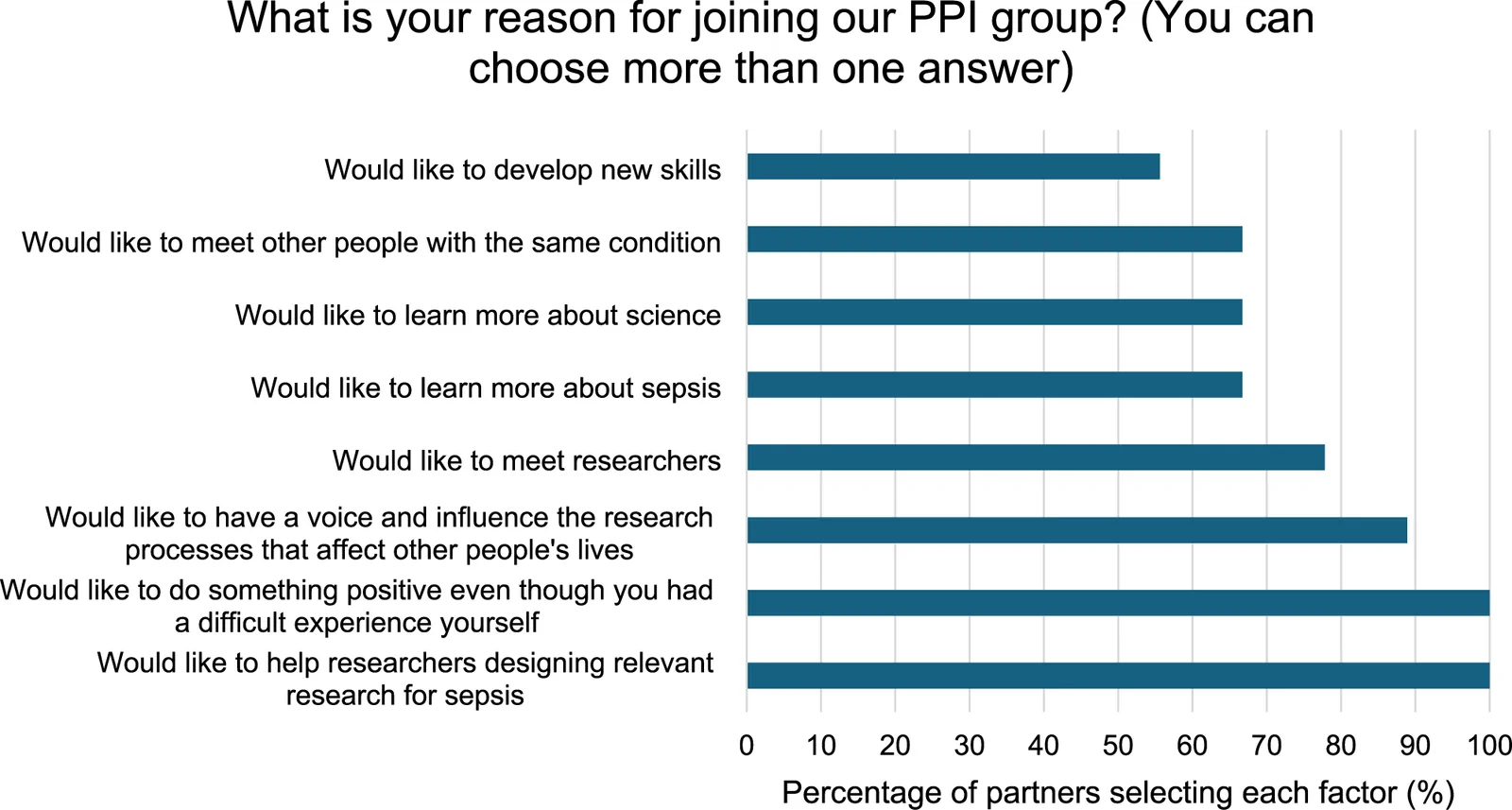
Partners’ reasons for joining the PPI group (*n* = 9)

The predominant reason for patient and public partners joining our PPI initiative was to turn their illness experience into something positive for society (Fig. 5). There was an overwhelming desire to help others in a similar position and to support researchers in understanding sepsis better. Most partners rated their own knowledge about sepsis as ‘good’ or ‘very good’ before joining the project but were keen to learn more about the condition and meet others who had been through similar experiences. Several partners were keen to understand more about how research was conducted and wanted to develop new skills in reading or writing scientific information.

All partners were very encouraging and supportive of our research and several commented that they had not previously appreciated the amount of work required to conduct a genomics study or to make a grant application.

At the end of the first year, all partners reported a high overall satisfaction with the process via individual emails to AL, and almost all (11/14) volunteered to remain patient and public partners for another year. All researchers reported a high overall satisfaction with the process, again via individual communication with AL.

The following (Table 1) shows several procedural challenges encountered by the group during the PPI initiative and suggestions of solutions to overcome them. The four main categories of challenges were: [1] administrative burden; [2] knowledge gaps; [3] emotional burden; and [4] lack of continuity.

**Table 1:** Procedural challenges encountered and solutions

Challenge	Solution
**Administrative burden** - Lack of researcher time - Lack of infrastructure to support PPI (including administrative support, appropriate remuneration claim forms)	Employment of PPI coordinators or points of contact within the group, with allocated time for PPI work Sharing experiences and materials with other groups or producing master copies that can be used throughout a department Seeking advice from experienced PPI coordinators or groups
**Knowledge gaps** - Lack of lay materials available to explain scientific concepts or procedures such as genome-wide association study - Wide heterogeneity in public understanding of disease and research - Gap between scientific knowledge and ‘lived experience’ (e.g. many researchers were well versed in the mechanisms of sepsis but not how these affected patients’ lived experiences)	Creating open-access educational materials Supporting the creation of a range of audiovisual educational tools for public education in science Participating in public engagement and seeking advice from public engagement departments within organisations Providing a safe space during PPI meetings for partners to share their lived experiences
**Emotional burden** - Emotional responsibility for patient and public partners (e.g. discussion about sepsis brought back many awful memories for partners) - Emotional burden on researchers – pressure to achieve numerous outcomes from short PPI sessions	Communication skills training Having a safety plan prepared before meetings (e.g. procedure if a patient or public partner reports feeling unwell) Setting clear aims for the PPI initiative Providing and following written agendas for meetings
**Lack of continuity** - Researchers on short term contracts, changing facilitators depending on availability	Involve more than one person during the recruitment process Ensure patient and public partners are aware of staff changes and always have an identified contact person within the group
**Losing engagement** - 3 patient and public members dropped out during the first year of the project. - There are long periods in between PPI related activities due to the nature of research work (e.g. awaiting result of a grant application)	Maintaining regular contact even when active PPI participation is not required

## Discussion

This study describes a successful pilot and launch of a PPI initiative for a discovery translational research group based on best practices and quality improvement principles. We found that partners were most motivated by a desire to do something positive despite their individual difficult experiences and to help researchers understand sepsis better. Patient and public partners felt that remuneration and acknowledgements were the least important aspects of their involvement. Patient and public partners were highly satisfied with the PPI process and learned a lot about the research process and about the genomic principles underlying sepsis. We observed that patient and public partners partners were able to join a grant steering group, help develop plain language summaries and materials for our study, and advise the group on future research directions and improving research processes, for example obtaining follow-up samples after patients were discharged home from hospital. We feel that the definition of PPI by NIHR was appropriate for the context in which it was used here.

Our findings are similar to previously reported researchers’ and patients’ attitudes toward PPI [20, 21] that developing and launching a PPI initiative for any group requires a significant investment of time and labour (both physical and emotional) but can be very beneficial if done well. Other reports on the use of PPI in research have highlighted the importance of good communication and setting clear expectations [22], which we paid particular attention to when designing our PPI programme.

A scoping review of public engagement in preclinical laboratory research identified 30 primary studies, with the majority engaging patients at priority setting and education stages [13]. The most frequent benefit identified as mutual learning opportunities. Barriers noted included knowledge gap, communication and limited opportunity for collaboration. Our work also identified the challenge of the knowledge gap, together with emotional burden for participants, administrative burden and continuity/maintain engagement. We have highlighted these and recommendationtions for how they could be addressed (Table 1).

An important outcome of our work with the PPI group were the patient and public partners partners’ opinions on which areas within sepsis research should be prioritised. Many of the group felt it was important to research the ‘unseen’ parts of the patient journey, such as recovery after leaving hospital. This mirrored findings from previous qualitative research of former critical care [23, 24] or Covid-19 patients [25], who have reported experiencing difficulties transitioning from being in a critical care setting to going home not fully recovered with limited opportunities for follow-up; worse in those who were severely ill but were not hospitalised in the case of Covid [25]. While this may be a result of survivorship bias in PPI recruitment, our PPI discussions reminded the researchers that the acute phase of severe infection and sepsis is only a relatively short part of individuals’ experience of the condition.

The PPI group also made important contributions to the clinical component of our larger studies, such as Sepsis Immunomics. The clinical components usually involved recruiting participants in the acute phase of illness and obtaining consent to perform repeated assessments which included blood tests and clinical information through the course of their illness and recovery. Most of the PPI group recounted similar experiences to other qualitative reports of patients experiencing sepsis: feelings of uncertainty [26], being too ill to take in any information, themselves and their families trying to process a large amount of information about their condition and the disease [27], and being in a rapidly evolving situation [28]. We had many discussions about the practicalities of recruiting participants to studies like ours in this setting, and the PPI group were able to help us develop patient information leaflets for our study that conveyed information about the study more clearly, alongside signposts toward useful resources to support patients through their illness, such as links to the Sepsis Trust. Long-term follow-up sample acquisition was also an interesting area of discussion, and the PPI group were able to provide advice about the reasons why participants often became lost to follow-up after hospital discharge, including difficulties mobilising, sleep disruption, and inability to drive while recovering from their illness.

The main strength of this study was the evidence-based approach to developing the PPI initiative and the use of quality improvement principles (plan, do, check, act cycle) to fine-tune processes. This led to a high overall satisfaction with the process from researchers and partners and the formation of a longer-term network.

The main weakness of our PPI initiative was selection bias in recruitment of PPI partners. The online advertisement for recruitment and use of online communication platforms for PPI meant that only individuals with access to the technological capability and devices, and the time to do so, could participate in the PPI initiative. All materials were written in the English language, thereby excluding people whose first language is not English. Patients who experienced the worst symptoms and worst ongoing quality of life might have been too unwell to participate.

## Conclusions

PPI is a valuable and useful initiative even in discovery translational research groups, leading to important outputs. More support is required to perform high-quality PPI in a discovery research setting. Future research should aim to investigate the impact of PPI group selection bias on research endeavours and identify the optimal method of advertising PPI opportunities, particularly in diverse demographic groups; and ways of measuring PPI impact on research.

## Data Availability

Data sharing is not applicable to this article as no datasets were generated or analysed during the current study.

## Abbreviations

PPIE: Patient and public involvement and engagement
PPI: Patient and public involvement
NIHR: National Institute of Health and Care Research
